# Co-Activation, Estimated Anterior and Posterior Cruciate Ligament Forces, and Motor Unit Activation Strategies during the Time Course of Fatigue

**DOI:** 10.3390/sports6040104

**Published:** 2018-09-21

**Authors:** Cory M. Smith, Terry J. Housh, Ethan C. Hill, Joshua L. Keller, Glen O. Johnson, Richard J. Schmidt

**Affiliations:** Department of Nutrition and Health Sciences, University of Nebraska-Lincoln, 110 Ruth Leverton Hall, Lincoln, NE 68583-0806, USA; thoush1@unl.edu (T.J.H.); Ethan.Hill@unl.edu (E.C.H.); Jkeller@unl.edu (J.L.K.); gojohnson10@gmail.com (G.O.J.); Rschmidt1@unl.edu (R.J.S.)

**Keywords:** ACL, PCL, isokinetic, electromyography, mechanomyography, coactivation, vastus lateralis, biceps femoris

## Abstract

This study aimed to combine co-activation as well as anterior and posterior cruciate ligament force estimations with the motor unit activation strategies employed by the primary muscles that are involved in the movement at the knee joint. Fourteen male subject performed 25 maximal concentric isokinetic leg extension muscle actions at 120 s^−1^. Electromyographic and mechanomyographic signals from the vastus lateralis and bicep femoris, as well as force, were used to measure co-activation, and estimated anterior and posterior ligament forces during the time course of fatigue. There were decreases in quadriceps force and increases in hamstring force during the 25 leg extensions. The posterior cruciate ligament force was greater than the anterior cruciate ligament force during each leg extension. Both the posterior and anterior cruciate ligament forces decreased during the 25 leg extensions. Each muscle indicated unique neuromuscular responses, which may explain the decreases in quadriceps force and increases in the hamstring force. The combination of anterior and posterior cruciate ligament force estimation and motor unit activation strategies helped to provide a better understanding of the fatigue-related mechanism that was utilized to avoid injury and increase or maintain joint stability during the time course of fatigue.

## 1. Introduction

The anterior and posterior cruciate ligaments are primarily responsible for stability of the knee. The anterior cruciate ligament stabilizes the knee during rotational, medial, lateral, and anterior to posterior planes of motion [1]. The posterior cruciate ligament primarily stabilizes the knee in the anterior to posterior plane of motion [2]. The anterior cruciate ligament is smaller in size when compared to the posterior cruciate ligament and rotational injuries have been suggested to be more common than anterior to posterior knee injuries [2,3]. Since the anterior cruciate ligament is smaller, it can become damaged and present with more distinct and easily clinically diagnosed injures as compared to the posterior cruciate ligament [2,3]. In addition, the majority of both anterior and posterior cruciate ligament injuries occur during physical activities including dynamic sports and activities of daily living [1,2]. Thus, examining changes in the forces placed upon the anterior and posterior cruciate ligaments during the process of fatigue during exercise may provide useful information regarding the balance between maximal force production and maintain joint stability.

Currently, there are no methods to directly measure the forces applied to the ligaments of the knee while performing dynamic activities of daily living or sports. Thus, the forces that are applied to the anterior and posterior cruciate ligaments must be estimated while using mathematical models that include various physiological measures [4]. Many studies have created estimations of the forces applied to the ligaments of the knee during walking, running, and cutting movements, but few studies have examined these ligament forces during isokinetic movements, which are common in research, physical therapy, and athletic training [4,5,6]. Isokinetic testing, which allows for control of the velocity and direction of movement, can provide a method for simultaneously examining the forces (or torque) that are associated with the anterior and posterior cruciate ligaments under controlled conditions [4,7]. In addition, the studies that aimed to quantify the forces placed on the ligaments of the knee during isokinetic muscle actions often used computer simulated models, which do not combine both real-time neuromuscular responses that are associated with the agonist and antagonist muscles forces [4,5,6]. That is, computer simulated models do not incorporate electromyographic signal from each subject, which has been reported [4] to improve the estimation of ligament forces.

During an isokinetic leg extension, the primary agonist is the vastus lateralis (VL), which pulls on the lateral quadriceps tendon to the patella, via ligamentum patellae into the tubercle of the tibia that drives the tibia out and upwards causing extension [8]. The primary antagonist during an isokinetic leg extension is the biceps femoris (BF), which pulls on the styloid process of the head of fibula, lateral collateral ligament, and lateral tibial condyle that pulls the tibia inward and back causing flexion [8]. The pull of the antagonist muscle against the agonist muscles is termed co-activation [9,10,11]. Co-activation affects net force production and gives insight to the amount of counter-pull forces that are placed on the joint during the muscle action [9,10,11]. The level of counter-pull may be estimated by measuring the co-activation by electromyographic (EMG) amplitude values of the antagonist muscle and can be used to examine the net forces of the knee [9,10,11]. That is, the greater the EMG amplitude of the antagonist, the greater the co-activation, and the greater the counter-pull forces that are being placed on the joint. The EMG amplitude is used to measure the level of co-activation forces because antagonist forces cannot be directly measured, but can be estimated by the EMG amplitude versus force relationship [12]. This relationship is obtained by simultaneously examining the amount of muscle activation from the antagonist muscle during the leg extension and estimating the force from the measured level of muscle activation. In addition, the level of muscle activation (EMG RMS) has also been used in equations to potentially increase the accuracy of the ligament force estimations during isokinetic leg extensions, which is used as the primary model in this study [4].

Electromyography and mechanomyography (MMG) allow for the simultaneous examination of muscle activation (EMG amplitude; root mean square (RMS)), motor unit action potential conduction velocity (EMG mean power frequency (MPF)), motor unit recruitment (MMG RMS), and motor unit firing rates (MMG MPF) [13,14,15,16]. It has been suggested that a fatigue-induced increase in the amplitude of the EMG signal reflects greater muscle activation [17], while a decrease in the frequency content reflects a slowing of motor unit action potential conduction velocity [17]. The MMG signal, however, has been described as the mechanical counterpart of the motor unit electrical activity as measured by EMG and quantifies the low-frequency oscillations of activated skeletal muscle fibers [18]. It has been suggested that, under some conditions, the amplitude of the MMG signal reflects motor unit recruitment [18] and the frequency content is qualitatively related to the global firing rate of unfused, activated motor units [18]. Therefore, it has been suggested that a fatigue-induced increase in MMG amplitude indicates greater motor unit recruitment, while a decrease in MMG frequency is associated with a decrease in firing rate [19]. Examining the neuromuscular responses over time can give insight to the motor unit activation strategies utilized to maintain force production and identify potential mechanisms utilized to increase or maintain joint stabilization [13]. In addition, it has been suggested [20] that co-activation from the hamstring muscles occurs during maximal isokinetic leg extensions and it plays a role in stabilizing the ligaments of the knee joint. During fatigue, there is often a decrease in stability of the knee joint that might stimulate mechanisms that increases co-activation to maintain joint stability and avoid injury [1,2,3]. Therefore, this study aimed to combine previously developed anterior and posterior cruciate ligament force estimations with the motor unit activation strategies employed by the primary muscles involved in the movement at the knee joint to better understand the potential fatigue-related mechanisms that are utilized to avoid injury and increase or maintain joint stability during the time course of fatiguing leg extensions. We hypothesized [13,14,15,16,20] that there would be fatigue-induced increases in co-activation from the BF accompanied by decreases in activation from the VL during the isokinetic leg extensions.

## 2. Material & Methods

### 2.1. Subjects

Fourteen men (mean ± SD age 22 ± 4.2 year; body mass 76.2 ± 9.4 kg; height 170.9 ± 5.2 cm) volunteered to participate in this study. All of the subjects were recreationally trained (greater than six-months of resistance training three times per week), and free from any musculoskeletal injuries or neuromuscular disorders [21]. This study was approved by the Institutional Review Board and was aligned with the Declaration of Helsinki [22]. All of the subjects signed a written informed consent and completed a health history questionnaire prior to participation.

### 2.2. Protocol

A warmup consisting of five to seven concentric isokinetic leg extension muscle actions were performed at approximately 50 to 70% of their perceived maximal effort. Following the warmup, each subject performed 25 maximal concentric isokinetic leg extension muscle actions at 120 s^−1^. All testing was performed on a Cybex II isokinetic dynamometer with a 90° range of motion. Electromyographic and MMG signals from the VL and BF, as well as force production values were simultaneously measured during each of the 25 repetitions. The subjects sat on a custom made platform which allowed simultaneous access to the VL and BF by a small pocket located under the BF muscle while seated.

### 2.3. Electromyographic, Mechanomyographic, and Force Signal Acquisition

**Figure 1:**
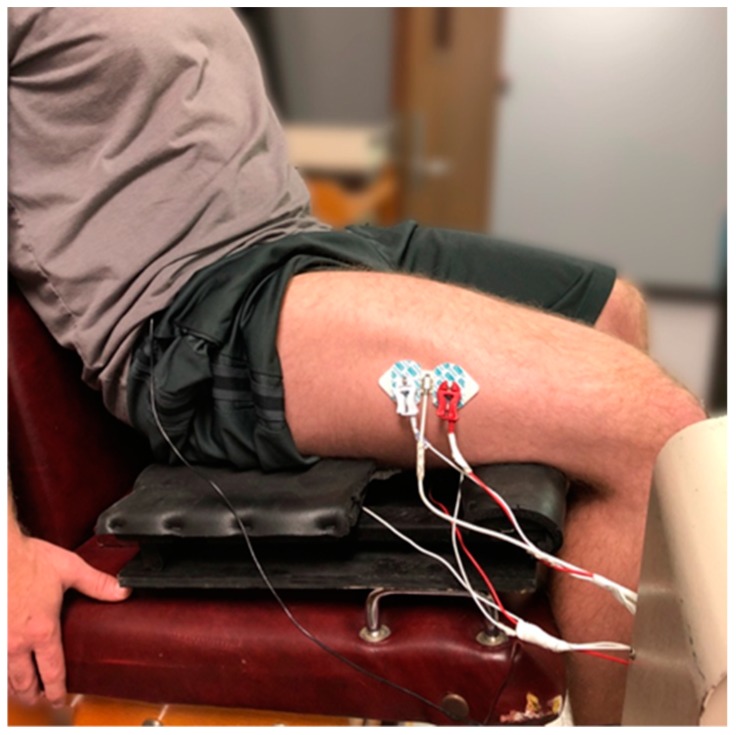
Depiction of the experimental setup. A notch is cut out under the thigh which allowed for the simultaneous measurement of electromyography and mechanomyography from the vastus lateralis and biceps femoris.

Bipolar surface electrode arrangements (Ag/AgCl, AccuSensor, Lynn Medical, Wixom, MI, USA) were placed on the VL and BF on the dominant leg (based on kicking preference) with an interelectrode distance of 30 mm. The skin was dry shaven, abraded, and cleaned with isopropyl alcohol prior to placing the electrodes. For the VL, the bipolar electrode arrangements were placed 66% of the distance between the anterior superior iliac spine (ASIS) and the lateral border of the patella, orientated at a 20° angle to approximate the pennation angle of the muscle fibers, and moved laterally 5 cm (Figure 1) [23,24,25]. For the BF, the bipolar electrode arrangement was placed 70% the distance from the ischial tuberosity and the lateral side of the popliteal cavity (Figure 1) [25]. A reference electrode was placed over the ASIS. The EMG signals were zero-meaned and bandpass filtered (fourth-order Butterworth) at 10–500 Hz. The MMG signal was measured while using triaxial accelerometers (EGAS-FT-10/V05, Measurement Specialties Inc., Hampton, VA, USA) placed between the bipolar electrode arrangements on the VL and BF. The MMG signals were zero-meaned and bandpass filtered (fourth-order Butterworth) at 5–100 Hz. Force was measured while using a low-profile pancake load cell (Honeywell Model 41, Morris Plains, NJ, USA) attached to the end of the lever arm behind the shin pad. All of the signals were simultaneously collected through a BioPac MP150 (BioPac System Inc., Goleta, CA, USA) at a sampling frequency of 10,000 Hz. The EMG RMS, EMG MPF, MMG RMS, and MMG MPF were calculated from the middle 33% of the EMG or MMG signal for each of the 25 repetitions (2475 Hz). All the signal processing was performed using custom programs written with LabVIEW software (Version 15.0, National Instruments, Austin, TX, USA).

### 2.4. Estimated Anterior and Posterior Cruciate Ligament Knee Forces

The fatigue-related changes in estimated anterior cruciate ligament forces and posterior cruciate ligament forces were calculated utilizing the models that were created by Zheng, Fleisig, Escamilla, Barrentine [4], and Delp [5]. The model of Zheng et al. [4] was specifically designed for estimating the forces placed on knee ligaments during exercise, including isokinetic muscle actions. Moment arm calculations were adapted from Herzog, Read [26] and Shelburne, Pandy [27] in accordance with the model of Zheng et al. [4]. The estimated anterior and posterior cruciate ligament forces were calculated for each repetition and individual forces were estimated while using Equation (1) with physiological cross-sectional area (PCSA) data from Wickiewicz et al. [28] (Equation (1)).

(1) $$F_{mi}=c_{i}k_{i}A_{i}q_{mi}EMG_{i}Peak\;Force_{i}$$

$F_{mi}$ represents the individual muscle forces utilized in Equation (2) for the determination of the anterior and posterior cruciate ligament forces, $c_{i}$ was a correction coefficient that was designed to minimize muscle force estimation errors [4,26], $k_{i}$ was the muscle force-length factor defined as the function of the knee angle [4,28], $A_{i}$ was the PSCA factor determined utilizing the model of Zheng et al. [4,28] for the VL and BF muscles, $q_{mi}$ was the peak force per PCSA for the VL and BF, $EMG_{i}$ was the amplitude of the EMG signal, and $Peak\;Force_{i}$ was the force applied at the lever arm. The regression equations of Herzog, Read [26] and adapted by Zheng et al. [4] were used to estimate the force coefficients for the anterior cruciate ligament (*F_acl_*), posterior cruciate ligament (*F_pcl_*), and tibiofemoral force (*F_tf_*) for the estimation of the anterior and posterior cruciate ligament forces during isokinetic muscle actions. The equations that were proposed by Zheng et al. [4] and Herzog, Read [26] utilized the velocity of the muscle action (120 s^−1^ in the current study), joint angle at peak force (typically between 110 to 130° with 180° being full extension), and PF*_t_* being the total peak force.

(2) $$F_{tf}+F_{pcl}+F_{ACL}=PF_{t}-\sum_{i=1}^{n}F_{mi}$$

### 2.5. Calculation of Antagonist Force

The estimations of the antagonist forces during the leg extension muscle actions were determined by using a linear EMG RMS versus force relationship. Each subject performed randomly ordered, step isometric leg flexion muscle actions at rest, 20, 40, 60, 80, and 100% maximal voluntary isometric contraction (MVIC). Electromyographic RMS was measured from the BF during each step muscle action. The EMG RMS values and the absolute force values for each individual subject were then used to develop a linear regression equation. Each subjects’ linear regression equation allowed for the estimation of force for a given EMG RMS value from the BF. Thus, the EMG RMS values that were obtained from the BF (co-activation) of each subject during the 25 maximal leg extension muscle actions were entered into to their corresponding linear regression equation to estimate the antagonist forces.

### 2.6. Statistical Analysis

The time course of changes in normalized neuromuscular responses (normalized to the initial repetition) for EMG RMS, EMG MPF, MMG RMS, and MMG MPF values were analyzed with one-way repeated measures ANOVAs (1 × 25 (Repetitions: 1 to 25)) with post-hoc Student Newman-Keuls tests being performed when appropriate to identify the time-points at which these values became different than the initial values [29].

The time course of changes in leg extension peak force and antagonist forces were analyzed by performing a one-way repeated measures ANOVA (1 × 25 (Repetitions: 1 to 25)) with post-hoc Student Newman-Keuls tests when appropriate [29].

A 2 (Estimated Anterior Cruciate Ligament Force and Posterior Cruciate Ligament Force) × 25 (Repetition: 1 to 25) repeated measures ANOVA was performed to compare the ligament forces during the isokinetic muscle actions. Follow-up one-way repeated measures ANOVAs (Repetition: 1 to 25) with post-hoc Student Newman-Keuls tests were performed when appropriate. The Student Newman-Keuls test was chosen for the post-hoc analyses, because it is designed to analyze the time course of changes in repeated measures variables [30]. The η${}_{p}^{2}$ effect size of 0.01 is considered small, 0.09 medium, and 0.25 large. An alpha of *p* ≤ 0.05 was considered to be statistically significant for all statistical analyses (SPSS Version 22.0, IBM, Armonk, NY, USA).

## 3. Results

### 3.1. Leg Extension Quadriceps and Estimated Hamstring Forces

**Figure 2:**
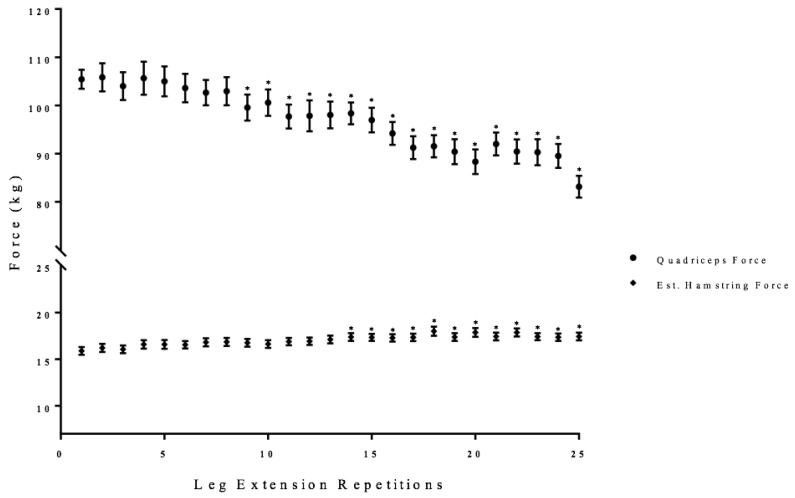
Time course of changes (mean ± SD) in quadriceps and hamstring forces during the fatiguing protocol. * Denotes significantly difference than the initial repetition.

There was a significant one-way repeated measure ANOVA for quadriceps force (*p* < 0.01, η${}_{p}^{2}$ = 0.37) that decreased from the initial repetition from 9 to 25 of the total leg extension repetitions (Figure 2).

There was a significant one-way repeated measure ANOVA for hamstrings force (*p* < 0.01, η${}_{p}^{2}$ = 0.36) that increased from the initial repetition from 14 to 25 of the total leg extension repetitions (Figure 2).

### 3.2. Neuromuscular Responses

**Figure 3:**
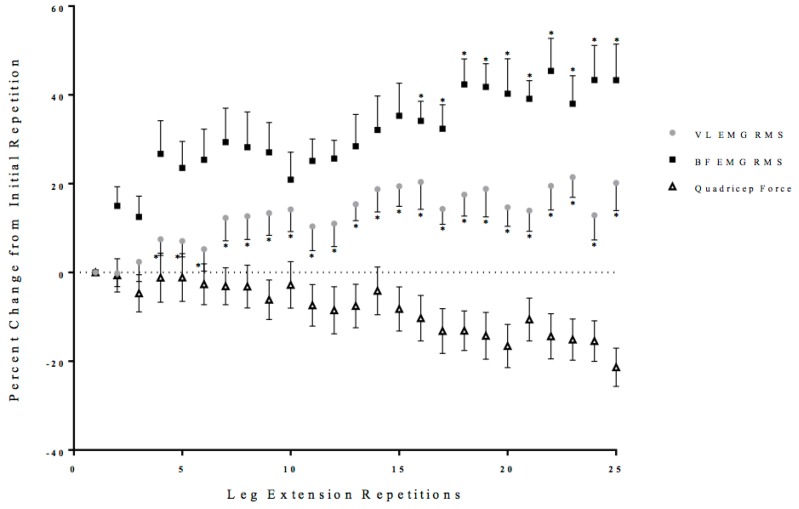
Time course of changes (mean ± SD) in the electromyographic amplitude (EMG RMS) from the vastus lateralis (VL) and biceps femoris (BF), as well as quadriceps force during the fatiguing protocol. * Denotes significantly different than the initial repetition.

Two separate, one-way repeated measure ANOVAs (Time: 1 × 25) were performed on the EMG RMS values from the VL and BF with post-hoc Student Newman-Keuls tests. There were significant one-way repeated measure ANOVAs from the VL (*p* = 0.02, η${}_{p}^{2}$ = 0.26) and BF (*p* < 0.01, η${}_{p}^{2}$ = 0.36), which increased from the initial repetition from repetition 4 to 25 and repetition 16 to 25 of the leg extension repetitions, respectfully (Figure 3).

**Figure 4:**
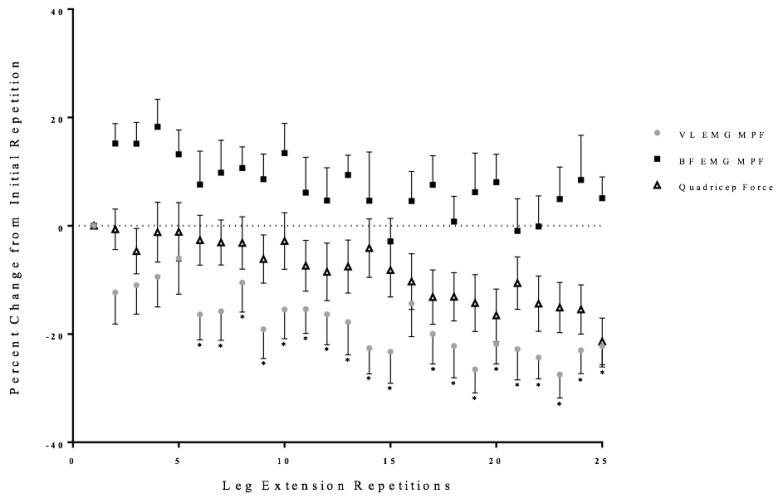
Time course of changes (mean ± SD) in the electromyographic frequency (EMG MPF) from the vastus lateralis (VL) and biceps femoris (BF), as well as quadriceps force during the fatiguing protocol. * Denotes significantly different than the initial repetition.

Two separate, one-way repeated measure ANOVAs (Time: 1 × 25) were performed on the EMG MPF values from the VL and BF with post-hoc Student Newman-Keuls tests. There was a significant one-way repeated measure ANOVA from the VL (*p* < 0.01, η${}_{p}^{2}$ = 0.35) that decreased from the initial repetition from repetition 6 to 25 of the leg extension repetitions (Figure 4). There was no significant one-way repeated measure ANOVA for the BF (*p* = 0.20, η${}_{p}^{2}$ = 0.11) (Figure 4).

**Figure 5:**
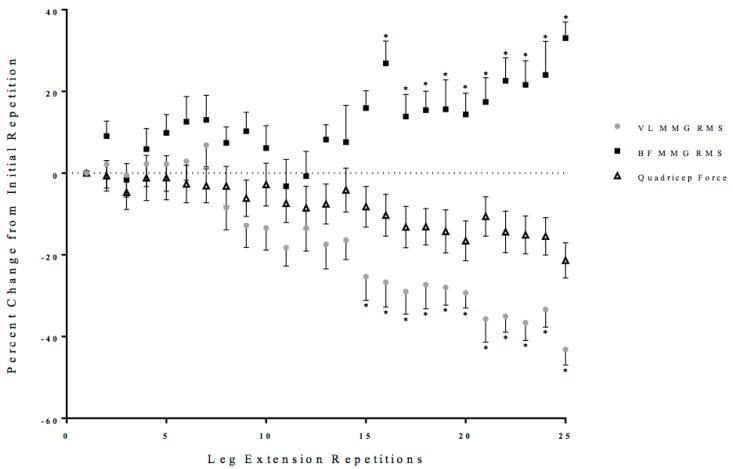
Time course of changes (mean ± SD) in the mechanomyographic amplitude (MMG RMS) from the vastus lateralis (VL) and biceps femoris (BF), as well as quadriceps force during the fatiguing protocol. * Denotes significantly different than the initial repetition.

Two separate, one-way repeated measure ANOVAs (Time: 1 × 25) were performed on the MMG RMS values from the VL and BF with post-hoc Student Newman-Keuls tests. There was a significant one-way repeated measure ANOVA from the VL (*p* < 0.01, η${}_{p}^{2}$ = 0.43) that decreased from the initial repetition from repetition 15 to 25 of the leg extension repetitions (Figure 5). There was a significant one-way repeated measure ANOVA from the BF (*p* = 0.04, η${}_{p}^{2}$ = 0.21), which increased from the initial repetition from repetition 16 to 25 of the leg extension repetitions (Figure 5).

**Figure 6:**
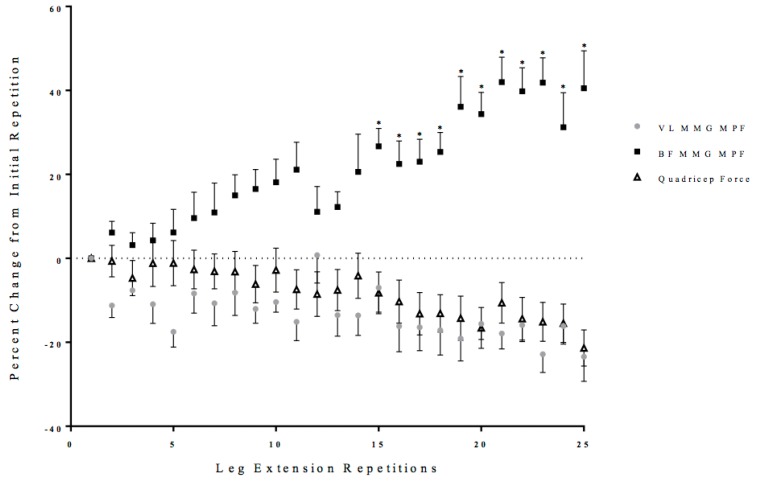
Time course of changes (mean ± SD) in the mechanomyographic frequency (MMG MPF) from the vastus lateralis (VL) and biceps femoris (BF), as well as quadriceps force during the fatiguing protocol. * Denotes significantly different than the initial repetition.

Two separate, one-way repeated measure ANOVAs (Time: 1 × 25) were performed on the MMG MPF values from the VL and BF with post-hoc Student Newman-Keuls tests. There was no significant one-way repeated measure ANOVA for the VL (*p* = 0.14, η${}_{p}^{2}$ = 0.09) (Figure 6). There was a significant one-way repeated measure ANOVA from the BF (*p* < 0.04, η${}_{p}^{2}$ = 0.19) that increased from the initial repetition from repetition 15 to 25 of the total leg extension repetitions (Figure 6).

### 3.3. Estimated Anterior and Posterior Cruciate Ligament Force 

**Figure 7:**
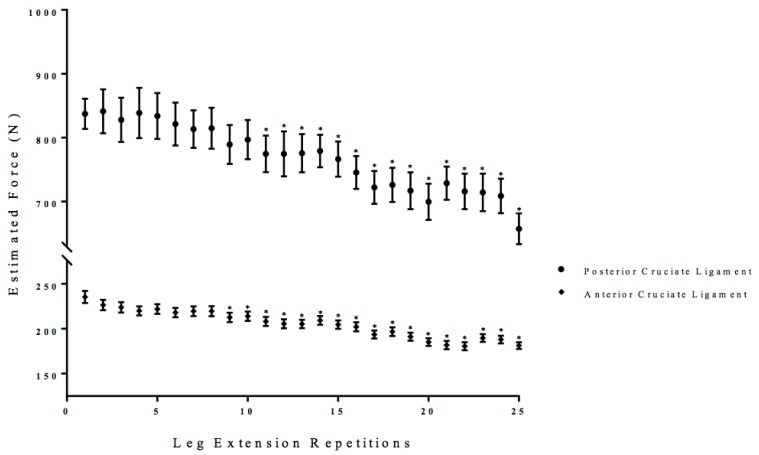
Time course of changes (mean ± SD) in the estimated posterior cruciate ligament and anterior cruciate ligament forces during the fatiguing protocol. * Denotes significantly different than the initial repetition.

There was a significant interaction (*p* < 0.01, η${}_{p}^{2}$ = 0.38) for the estimated anterior and posterior cruciate ligament forces. Follow-up analyses indicated that posterior cruciate forces were greater than anterior cruciate forces for each repetition (*p* < 0.01, η${}_{p}^{2}$ = 0.40). Two separate, follow-up one-way repeated measure ANOVAs (Time: 1 × 25) were performed on the anterior and posterior cruciate ligament forces with post-hoc Student Newman-Keuls tests. There was a significant one-way repeated measure ANOVA for the anterior cruciate ligament force (*p* < 0.01, η${}_{p}^{2}$ = 0.35) and posterior cruciate ligament (*p* < 0.01, η${}_{p}^{2}$ = 0.39) that decreased from the initial repetition from 9 to 25 and 11 to 25 of the total leg extension repetitions (Figure 7).

## 4. Discussion

The current study was designed to simultaneously examine neuromuscular responses and estimated anterior and posterior cruciate ligament forces during the time course of fatigue to examine the balance between force decrement during fatigue, anterior and posterior cruciate ligaments, and co-activation. In this study, both anterior and posterior cruciate ligament forces, as well as quadriceps force, decreased as a result of the fatiguing leg extensions. These decreases in forces are likely attributed to fatigue-related neuromuscular changes and an increase in co-activation that resulted in a counter-pull during the leg extensions.

Maximal quadriceps force was maintained until repetition 9, when it began to decrease significantly from the initial repetition (Figure 2). The maintenance of quadriceps force during the initial repetitions may be explained by the neuromuscular responses derived from the VL and BF. The neuromuscular responses from the BF suggested that the counter-pull from the antagonist muscle did not impact maximal quadriceps force during the first nine repetitions (Figure 2). The neuromuscular responses from the VL, however, indicated that maximal muscle activation (EMG RMS) was not reached until approximately repetitions 4 to 7 (Figure 3). There were three primary mechanisms that may explain their initial increases in muscle activation (EMG RMS): (1) the subjects were not initially giving maximal effort; (2) a potential reserve capacity in strength; or, (3) motor unit synchronization [7,16]. It has been suggested that it takes approximately three to four repetitions to achieve maximal force production during maximal isokinetic or isometric leg extension [7]. The reserve capacity, however, is a theory that suggests the body retains a percentage of a muscles maximal strength as a protective mechanism for stressful or strenuous situation [16]. It has been suggested that fatigue and repetitive maximal muscle actions to provide sufficient stress and afferent stimulus of the nervous system to illicit the use of this reserve capacity [13,28,31]. Motor unit synchronization, however, is a theory that suggested the firing of the motor units could become synchronized to optimize force production that causes an increase in EMG amplitude [32,33,34]. Thus, the initial increases in muscle activation (EMG RMS) from the VL and the ability to maintain force during the first nine repetitions may be explained by the effort of the muscle action, reserve capacity, or motor unit synchronization. 

At repetition 9, quadriceps force began to decrease and it was accompanied by a decrease in the anterior cruciate ligament force (Figure 2 and Figure 7). Muscle activation (EMG RMS) from the quadriceps reached maximal values at repetition 10, but was insufficient to maintain quadriceps force (Figure 3). It is likely that there was a fatigue-induced build-up of metabolic byproducts in the quadriceps that began to influence the VL beginning at approximately repetition 6, as indicated by the decrease EMG MPF (Figure 4). From repetitions 11 to 13, there was no change in muscle activation (EMG RMS) of the VL and decrease in EMG MPF that potentially contributed to the decrease in force production (Figure 3 and Figure 4). In addition, there were decreases in the anterior and posterior cruciate ligament forces which were likely due to the decreased quadriceps force placing lower forces on both the anterior and posterior cruciate ligaments. 

Unlike the first half of the 25 maximal leg extensions, repetitions 14 to 16 were characterized by increases in co-activation, as reflected from the EMG RMS from the BF, and decreases in EMG MPF and MMG RMS from the VL (Figure 3, Figure 4, Figure 5 and Figure 6). Specifically, there were increases in BF muscle activation (EMG RMS), motor unit recruitment (MMG RMS), and global motor unit firing rates (MMG MPF) during the 25 maximal leg extension muscle actions, which indicated a greater co-activation (Figure 3, Figure 4, Figure 5 and Figure 6). In addition, there was an increase in hamstring force, as indicated by the increased BF activation, that resulted in a counter-pull action during the leg extensions, resulting in a further decrease in quadriceps force production (Figure 2). From repetitions 17 to 25, there was continued decrease in MMG RMS from the VL as well as increases in co-activation from the BF. In addition, there were continued decreases in quadriceps force and anterior and posterior cruciate ligament force, as well as increases in hamstring force (Figure 2, Figure 3, Figure 4, Figure 5, Figure 6 and Figure 7).

The increases in the hamstring forces, as indicated by the BF electromyography activation, may be explained by the neuromuscular responses from the BF (Figure 2, Figure 3, Figure 4, Figure 5, Figure 6 and Figure 7). Specifically, the BF exhibited increases in muscle activation (EMG RMS), motor unit recruitment (MMG RMS), and motor unit firing rates (MMG MPF) (Figure 2, Figure 3 and Figure 4). There were no changes in EMG MPF, which suggested that the BF was not fatigued and, likely, did not have a fatigue-related buildup of metabolites during the 25 maximal isokinetic leg extensions (Figure 4) [35,36]. Thus, during the maximal isokinetic leg extensions there were increases in the hamstring forces that were likely due to increases in muscle activation, motor unit recruitment, and motor unit firing rates.

The decreased force production from the quadriceps, but increased hamstring forces during the fatiguing leg extensions were in agreement with previous studies that showed decreased leg extension force associated with increased co-activation of the BF [9,10,11,20]. Although the exact degree of influence that BF co-activation has on quadriceps force production is uncertain due to the inability to simultaneously measure agonist and antagonist forces, a linear muscle activity versus force relationship similar to the current study are often assumed for co-activation analyses [4,12,37]. The increased hamstring force, indicated by the BF, suggested that the decrease in quadriceps force may partially be explained by the hamstring muscle acting as a counter-pull during the leg extensions (Figure 2).

The estimated posterior cruciate ligament force was greater than the estimated anterior cruciate ligament force during each maximal isokinetic leg extension and both ligament forces decreased during the fatiguing isokinetic leg extensions (Figure 7). In addition, the initial estimated posterior cruciate ligament force and anterior cruciate ligament force were similar to those that were reported by Zheng et al. [4] who utilized a similar model and were examined during maximal isokinetic leg extensions. The lower anterior cruciate ligament force when compared to the posterior ligament force can be explained by the mode of exercise. That is, isokinetic leg extensions are performed in a seated position that stabilize the torso, quadriceps, knee joint, and ankle, which results in limited rotational forces placed on the knee joint [2,3]. The anterior cruciate ligament receives peak forces during rotational or lateral applications of forces, such as cutting in soccer or football [1,38]. The high degree of rotational stability associated with isokinetic leg extensions limited the forces applied to the anterior cruciate ligament during maximal isokinetic leg extensions when compared to the forces that were observed during dynamic sports and resistance training [1,2,3,4,38].

The posterior cruciate ligament is aligned with the center of rotation and is considered to be a fundamental stabilizer of the knee joint [3]. In addition, the posterior cruciate ligament is more robust than the anterior cruciate ligament and is capable of sustaining greater forces without injury [3]. The primary function of the posterior cruciate ligament is to maintain posterior displacement of the tibia, and, therefore, has the highest forces placed upon it during anterior and posterior force application [2,3]. The knee joint angle during concentric isokinetic leg extension move from the posterior to anterior position. During this posterior to anterior movement, the posterior cruciate ligament is primarily relied upon for stabilization of the knee joint. When performing isokinetic leg extension, there is an increased stability of rotational forces that are associated with the setup of the isokinetic dynameter. That is, the isokinetic dynamometer allows for the subjects to be seated with their thigh supported and ankle strapped to the lever arm which provides greater stability than a movement such as a squat. The increased rotational stability during isokinetic leg extensions may have resulted in lower anterior cruciate ligament forces and placed greater forces on the posterior cruciate ligament due to the majority of the required stability being in the posterior to anterior plane of motion.

The decreases in anterior and posterior cruciate ligament forces, accompanied by decreases in quadriceps force and increases in hamstring force, might represent a protective mechanism against injuries during dynamic fatiguing muscle actions. That is, increases in laxity of the anterior and posterior ligaments have been associated with hyperextension, hyperflexion, anterior tibial displacement, posterior tibial displacement, and greater instability of the knee joint [1,3,6]. In addition, the decreases in the quadriceps force with increases in hamstring force indicated that absolute maximal force production may be modulated by afferent feedback to increase stability of the knee joint [1,2,3]. This efferent feedback may signal for increases in stability, which results in a reduction in force production and lowers the forces applied to the anterior and posterior cruciate ligament in an attempt to avoid injury, especially when there has been exercise-related increases in the ligaments laxity. Hypothetically, had the hamstrings not increased in muscle activation and provided a counter-pull there would be an increased risk of hyperextension or posterior tibial displacement which are both primarily posterior cruciate ligament injuries [3]. Thus, fatigue-related decreases in quadriceps force and increases in hamstring force during fatiguing isokinetic leg extensions resulted in decreased anterior and posterior cruciate ligament forces, which may reflect protection mechanisms against injury to the knee joint from the effects of exercise and temperature-related laxity.

## 5. Conclusions

In conclusion, there were decreases in quadriceps force with increases in hamstring force, indicated by the BF, during the fatiguing isokinetic leg extensions. The decreases in quadriceps force were accompanied by decreases in motor unit recruitment (MMG RMS), and motor unit action potential conduction velocity (EMG MPF), but no changes in motor unit firing rates (MMG MPF) from the VL. The increases in hamstring force were accompanied by increases in muscle activation (EMG RMS), motor unit recruitment (MMG RMS), and motor unit firing rates (MMG MPF) from the BF. In addition, the posterior cruciate ligament force was greater than the anterior cruciate ligament force during each maximal isokinetic leg extension. Both the posterior and anterior cruciate ligament forces, however, decreased during the fatiguing isokinetic leg extensions. This was likely due to the increased stability of rotational forces during the isokinetic leg extensions when compared to the stability in the posterior to anterior plane of motion. The decreases in quadriceps force and increases in hamstring force during the fatiguing isokinetic leg extensions accompanied by decreased anterior and posterior cruciate ligament forces may reflect a protective mechanism against injury from the effects of exercise-related laxity during maximal isokinetic leg extensions. Thus, electromyography, mechanomyography, and force can be utilized to evaluate the motor unit activation strategies, anterior and posterior cruciate ligament forces, and the influence of co-activation during the process of fatigue.
